# Are There More HER2 FISH in the Sea? An Institution’s Experience in Identifying HER2 Positivity Using Fluorescent In Situ Hybridization in Patients with HER2 Negative Immunohistochemistry

**DOI:** 10.1245/s10434-023-14439-7

**Published:** 2023-11-07

**Authors:** Camille Suydam, Fairouz Chibane, Nicole Brown, Madeleine Schlafly, Alicia H. Arnold, Intisar Ghleilib, Melissa Easley, Joseph White

**Affiliations:** 1 Department of Surgery, Eisenhower Army Medical Center, Fort Gordon, GA USA; 2 Department of Surgery, Medical College of Georgia at Augusta Health, Augusta, GA USA; 3 Department of Pathology, Medical College of Georgia at Augusta Health, Augusta, GA USA

**Keywords:** HER2, FISH, IHC, False negative, Reflex testing

## Abstract

**Background:**

Approximately 20% of breast cancers express HER2-positive receptors in the USA. HER2 receptor immunohistochemistry (IHC) staining with equivocal (2+) results commonly undergoes fluorescence in-situ hybridization (FISH) for further classification. Current guidelines do not recommend routine FISH testing in IHC-negative (0 or 1+) cases. This study investigates an institution that performs both IHC and FISH testing on all cases to identify the true HER2-positive rate.

**Patients and Methods:**

A retrospective chart review from 2015 to 2021 was conducted at an institution where both HER2 IHC and FISH testing were performed at the time of diagnosis for all invasive breast cancers. The rate of true HER2-positive patients was determined, and patient and tumor characteristics were further explored.

**Results:**

A total of 1835 invasive breast cancer cases were primarily treated at this institution. A total of 289 cases were HER2 positive on IHC and FISH testing (15.7%). An additional 38 cases were identified as HER2 negative on IHC, but reclassified as HER2 positive on reflex FISH testing. Total HER2 positive cases increased from 289 (15.7%) to 327 cases (17.8%) with reflex FISH testing.

**Conclusions:**

The additional HER2-positive cases after completing FISH testing on IHC-negative tumors suggests there may be a role for routine FISH testing in addition to standard IHC staining to determine HER2 status for breast cancer. The ethical, prognostic and even  benefits of a correct diagnosis outweigh the added expense of FISH testing.

In the USA, breast cancer is the most commonly diagnosed cancer and the second most common cause of cancer death in women.^1^ Breast cancer is a diverse disease with several biologic subtypes that have distinct behavior, each necessitating a different treatment. One of these subtypes is defined by an overexpression of the human epidermal growth factor receptor 2 (HER2) gene, previously called HER2/neu or ERBB-2. This gene plays a critical role in controlling epithelial cell growth, differentiation,^2,3^ and possibly angiogenesis.^4,5^ Approximately 15–20% of primary invasive breast cancers are HER2 positive.^6^ HER2 gene amplification is associated with a relatively poor prognosis with increased risk of recurrence and metastasis,^7^ but is also a predictive factor for response to anthracycline-based chemotherapy and treatment benefit with anti-HER2 therapies such as trastuzumab, lapatinib, and pertuzumab.^8^

Since the development of anti-HER2 therapy, outcomes for HER2-positive patients have improved significantly.^9,10^ The accurate identification of HER2 status and initiation of disease-specific therapy can decrease the risk of death and alter the prognosis of a patient who has HER2-positive breast cancer.^9^ HER2 status is assessed in all newly diagnosed, invasive, or recurrent breast tumors. However, the best method for determining the HER2 status continues to be a source of controversy. Standard methods involve immunohistochemistry (IHC) and fluorescence in situ hybridization (FISH). IHC is most often used as the initial HER2 screening test because of its low cost, availability, and ease of implementation.^11^ However, certain aspects of quality control, such as the timing and process of tissue fixation, can impact the accuracy of the test.^11,12^ Grading of IHC staining ranges from 0 to 3+, with an IHC grading of 3+ considered HER2 positive, 2+ considered HER2 equivocal, and 1+ or 0 considered HER2 negative. Current guidelines set forth by the American Society of Clinical Oncology and the College of American Pathologists recommend reflex FISH testing when the grade of IHC is 2+ (equivocal). FISH testing evaluates the number of HER2 gene copies within the nucleus using fluorescence labeled nucleic acid probes.^11^ The result is then interpreted on the basis of the HER2/centromere enumerator probe 17 (CEP17) ratio and HER2 copy number per the American Society of Clinical Oncology (ASCO)/College of American Pathologists (CAP) guidelines, last updated in 2018.^13^ Although FISH testing may not be as susceptible to the same errors as IHC, the testing method is more complicated and expensive.^12^

Reflex FISH testing in the setting of an IHC HER2 equivocal status has diagnosed a significant number of HER2 positive patients.^14^ However, the current guidelines do not recommend reflex FISH testing in the setting of a negative initial IHC staining.^15,16^ These guidelines are based on literature suggesting a concordance as high as 96% between IHC protein overexpression and FISH gene amplification.^12^ However, while concordance may be high, it is not perfect. Given the lack of data on reflex FISH testing in IHC 0 or 1+ patients, in this study we aimed to investigate the actual rate of HER2-positive tumors at our institution, on which we have routinely completed reflex FISH testing on IHC HER2-negative (1+ and 0) tumors since 2015. We also aimed to evaluate the outcomes and therapeutic implications of this reflex testing, thus identifying a potential protocol for breast cancer specimen processing.

## Patients and Methods

A retrospective chart review from 2015 to 2021 was conducted at a single institution where both HER2 IHC staining and reflex FISH testing were performed at the time of diagnosis for all invasive breast cancers. The same area of tumor was used for each test. Both of these tests were performed in-house, with results requiring agreement by two pathologists. Although not part of ASCO–CAP guidelines, this reflex FISH testing was made part of the testing protocol at our institution as a quality improvement initiative given multiple instances of IHC 2+ tumors not being recognized and reflex tested in a timely manner, leading to delays in appropriate management.

Prior to accessing the data, this study was determined to be exempt from Institutional Review Board (IRB) review by the Augusta University IRB. Patients with HER2-negative (0 or 1+) results on IHC with subsequent HER2-positive results on reflex FISH testing were included. Patients with HER2 equivocal (2+) or HER2 positive (3+) on initial IHC were excluded. Demographic data and pertinent patient history were extracted, including age, sex, race, weight, height, body mass index (BMI), smoking status, comorbidities (diabetes, hypertension), date of diagnosis, cancer subtype, cancer stage, treatment plans (surgical therapy, radiation, chemotherapy, endocrine treatment), and outcomes.

A cost analysis was performed by calculating the total cost of common chemotherapy regimens for HER2-negative and HER2-positive patients based on current estimations at our institution for a 60–70 kg patient. These estimations were obtained from the Augusta University Oncology Pharmacy Department and were based on price without insurance coverage.

## Results

### HER2 Status

A total of 1835 invasive breast cancer cases were primarily treated at this institution. A total of 289 cases were found to be HER2 equivocal or positive (2+ or 3+) on IHC testing with FISH confirmation (15.7%). An additional 38 specimens were classified as HER2 negative (0 or 1+) on IHC, but subsequently reclassified as HER2 positive on reflex FISH testing (2.1%). This increased the total rate of HER2 positive cases from 289 (15.7%) to 325 cases (17.8%).

### Patient and Tumor Characteristics

There were 38 patients who initially tested as HER2 negative on IHC (1+ or 0) but were subsequently found to be HER2 positive on reflex FISH testing. The majority of these patients were African American and between the ages of 40 and 65 years; most patients were non-diabetic, non-smokers, hypertensive, and obese (Table 1). The majority of tumors were invasive ductal carcinoma, with most being hormone receptor positive. The majority of tumors had a HER2/CEP17 ratio < 3.0 and a HER2 copy number < 6.0 Twenty-eight patients received either neoadjuvant or adjuvant anti-HER2 therapy (Table 2).

**Table 1:** Patient demographics

		Number (n = 38)
Race	Caucasian	13
	African American	24
	Hispanic	1
Smoking status	Current smoker	4
	Former smoker	13
	Never smoker	19
	Smokeless tobacco	2
Hypertension	Yes	21
	No	17
Diabetes	Yes	9
	No	29
Age (years)	< 40	4
	40–65	25
	> 65	9
BMI (kg/m^2^)	< 25	5
	25–29.9	15
	> 30	18

**Table 2:** Tumor characteristics and anti-HER2 treatment

		Number (n = 38)
Specimen source	Initial core biopsy breast	20
	Initial core biopsy lymph node	4
	Surgical excision breast	7
	Surgical excision lymph node	3
	Distant metastasis biopsy	4
Tumor Type/subtype	Ductal	29
	Ductal with lobular features	2
	Ductal/mucinous	1
	Ductal/micropapillary	1
	Ductal with atypical lobular hyperplasia/mucinous	1
	Lobular	1
	Neuroendocrine	1
	Poorly differentiated	2
Grade	1	6
	2	10
	3	12
	Unknown	10
Stage	I	11
	II	10
	III	3
	IV	14
Hormone status	ER+/PR+	18
	ER+/PR−	3
	ER−/PR+	4
	ER−/PR−	13
HER2/CEP17 ratio	< 2.0^a^	1
	2.0–2.9	26
	3.0–5.0	7
	> 5.0	4
HER2 copy number	< 4.0^a^	1
	4.0–5.9	17
	6.0–9.0	12
	> 9.0	8
Anti-HER2 treatment	Trastuzumab	17
	Trastuzumab and pertuzumab	13
	None	8

^a^Patients that fell in this category were tested prior to 2018 and considered HER2 positive based on 2013 ASCO–CAP guidelines

### Cost Analysis

The chemotherapy regimen appropriate for HER2-positive patients at our institution is estimated to cost US$274,615 for adjuvant therapy (Table 3) and US$404,491 for neoadjuvant therapy (Table 4). The chemotherapy regimen appropriate for HER2-negative patients at our institution is estimated to cost between US$18,532 (Table 5) and US$20,664 (Table 6).

**Table 3:** Chemotherapy regimen/cost for HER2 positive patients, adjuvant

Taxol and Herceptin (TH)		
Drug	One cycle	Treatment course
Paclitaxel (weekly)	$203	$2436 (12 doses)
Trastuzumab (Q3 weeks)	US$19,715 (loading dose) US$15,779 (maintenance)	$272,179 (1 loading dose + 16 maintenance doses)
Total	US$19,918 (loading) US$15,982 (maintenance)	$274,615

**Table 4:** Chemotherapy regimen/cost for HER2 positive patients, neoadjuvant

Taxotere, Carboplatin, Herceptin, and Perjeta (TCHP)			
Drug	First cycle	Second cycle	Treatment course
Docetaxel	$1428	$1428	$8568 (6 doses)
Carboplatin	$368	$368	$2208 (6 doses)
Trastuzumab (Q3 weeks)	$19,715 (loading dose)	$15,779 (maintenance)	$272,179 (1 loading dose + 16 maintenance doses)
Pertuzumab (Q3 weeks)	$13,504 (loading dose)	$6752 (maintenance)	$121,536 (1 loading dose + 16 maintenance doses)
Total	$35,015	$24,327	$404,491

**Table 5:** Chemotherapy regimen/cost for HER2− patients, option one

Dose-dense Adriamycin, dose-dense Cytoxin, and Taxol (ddAC–T)		
Drug	One cycle	Treatment course
Doxorubicin	US$284	$1136 (4 doses)
Cyclophosphamide	US$3740	$14,960 (4 doses)
Paclitaxel	US$203	US$2436 (12 doses)
Total	US$4227	US$18,532

**Table 6:** Chemotherapy regimen/cost for HER2− patients, option two

Taxol/Taxotere and Cytoxan (TC)		
Drug	1 cycle	Treatment Course
Docetaxel	US$1428	US$5712 (4 doses)
Cyclophosphamide	US$3738	US$14,952 (4 doses)
Total	US$5166	US$20,664

## Discussion

At our institution, an additional 2.1% of patients with invasive breast cancer were found to be HER2 positive due to routine reflex FISH testing. This data is concordant with the model-based study by Garrison et al., in which the authors concluded that an additional 2.27% of women would be found to be HER2 positive with reflex FISH testing after initial HER2 negative results via IHC. They also estimated that 4700 additional women would collectively gain 9016 life years and 8330 quality-adjusted life years due to correctly diagnosing HER2 status.^12^ Another study found that of 499 patients whose tumors had been previously classified as HER2 negative, 22 patients (4%) were ultimately reclassified as HER2 positive,^11^ suggesting that reflex testing may provide an even greater therapeutic benefit than Garrison et al. propose.

Interestingly, out of our 38 patients, there were some relatively rare histologic subtypes; this included one micropapillary subtype (2.6%), two mucinous subtypes (5.3%), and one neuroendocrine subtype (2.6%). While the exact prevalence of these subtypes is not well established, one recent study with a sample size of over 1000 patients found that ~ 0.02% of tumors demonstrated a micropapillary subtype, ~ 0.02% demonstrated a mucinous subtype, and < 0.01% demonstrated a neuroendocrine subtype.^17^ This suggests that IHC testing for HER2 may be less reliable for tumors that demonstrate rare histologic features. Also of note, the most common tumor grade among our 38 patients was grade 3 (12 patients), which may indicate that IHC testing is also less reliable for more aggressive tumors, or at least one′s index of suspicion for HER2 positivity should be increased in higher grade tumors.

It should be addressed that the majority of the tumors that were negative on IHC but positive on FISH had a HER2/CEP17 ratio < 3 and a HER2 copy number < 6. While all but two of these are still considered HER2 positive based on the updated 2018 ASCO–CAP guidelines (HER2/CEP17 > 2.0 and HER2 copy number > 4.0),^13^ they are relatively close to the cutoff. Regardless, they are still FISH positive and should therefore still be treated with anti-HER2 treatment as appropriate. This may even pose a stronger argument that FISH testing should be done routinely, as these cases with lower HER2/CEP17 ratios and HER2 copy numbers may be more easily missed on IHC.

While it should not be the sole factor and is of the least importance, cost effectiveness is a consideration when establishing new protocols or guidelines for diagnosing and treating breast cancer. Our cost analysis demonstrated a roughly ten-to-twenty-fold increase in the cost of chemotherapy for HER2-positive patients. This implies there is a substantial revenue loss for the treating institution if HER2-positive patients are not identified and treated appropriately, considering most patients are able to obtain insurance with a breast cancer diagnosis since the passing of the Affordable Care Act.^18^ In their model-based study, Garrison et al. addressed the cost effectiveness of correctly diagnosing HER2-positive breast cancer due to the clinical benefit of the addition of trastuzumab to adjuvant chemotherapy. Although a FISH test was more expensive than an IHC test (US$339 versus US$118), they found that the cost-effectiveness ratio of correctly diagnosing and treating HER2-positive breast cancer in a larger percentage of women is greater than the initial upfront cost of utilizing both tests.^12^ At our institution, the additional cost of adding reflex HER2 FISH testing was negligible compared with the revenue we obtained by appropriately treating the additionally identified HER2-positive patients. We found the average cost of the regimens most commonly used at our institution for HER2-positive patients to be US$274,615 for adjuvant therapy and US$404,491 for neoadjuvant therapy. In contrast, the cost of the regimens most commonly used at our institution for HER2-negative patients ranges from US$18,532 to US$20,664. While anti-HER2 therapy may not always be indicated in HER2 patients (as discussed below), it was indicated for the majority of our patients resulting in a substantial increase in revenue for our institution.

Besides the potential financial detriments of a false negative HER2 diagnosis, the ethical cost of providing an inaccurate diagnosis and inappropriate treatment to our patients with cancer cannot be measured in the monetary sense. The current National Comprehensive Cancer Network (NCCN) guidelines recommend that patients that are HER2-positive receive adjuvant trastuzumab for tumors > 1 cm, and that trastuzumab should also be considered on an individual basis for tumors < 1 cm, as the benefits and risks are not yet clear in this patient population; they also suggest that this should be more strongly considered in hormone receptor-negative patients. The NCCN also recommend considering the addition of pertuzumab for any HER2-positive patients with nodal disease.^19^ Indeed, some studies have demonstrated that dual blockade with trastuzumab and pertuzumab leads to significantly greater rates of survival in HER2-positive patients.^20,21^ Neoadjuvant anti-HER2 therapy is also becoming increasingly common.^22^ Patients who are treated neoadjuvantly with anti-HER2 therapy in addition to chemotherapy have been found to have a significantly improved pathologic complete response rate,^23,24^ which could potentially allow breast conservation therapy in patients not previously eligible^25^ and sparing of axillary lymph node dissection in initially node-positive patients.^25,26^ One of the main concerns with anti-HER2 therapy is the risk of cardiotoxicity of which a wide range of incidence has been reported,^20^ although the recent APHINITY trial reported < 1% of patients experienced a primary cardiac event.^21^ While the risk of adverse events should be kept in mind, the survival benefit of anti-HER2 therapy cannot be ignored.

One limitation of this study is the limited sample size of 38 patients. We also do not have information regarding the correlation between the amount of tumor seen in the core biopsy versus the final surgical specimen. Future studies that include multiple centers that routinely perform reflex FISH testing on all breast specimens would be helpful to assess for a more accurate representation of the true HER2-positive incidence. Future studies could also evaluate the possibility of eliminating IHC testing and relying solely on FISH testing for increased cost effectiveness. The correlation between specific tumor factors (such as rare histologic subtypes and advanced grade) and false negative HER2 results on ICH should also be explored further.

## Conclusions

The significant increase in rates of HER2 positivity after completing FISH testing on IHC 0 or 1+ suggests that there may be a role for routine FISH testing in addition to standard IHC staining to determine HER2 status for breast cancer. The financial, ethical, and prognostic benefits of a correct diagnosis outweigh the added expense of FISH testing.
